# Real-time spectrum estimation–based dual-channel speech-enhancement algorithm for cochlear implant

**DOI:** 10.1186/1475-925X-11-74

**Published:** 2012-09-24

**Authors:** Yousheng Chen, Qin Gong

**Affiliations:** 1 Department of Biomedical Engineering, Tsinghua University, Beijing, 100084, PR China

## Abstract

**Background:**

Improvement of the cochlear implant (CI) front-end signal acquisition is needed to increase speech recognition in noisy environments. To suppress the directional noise, we introduce a speech-enhancement algorithm based on microphone array beamforming and spectral estimation. The experimental results indicate that this method is robust to directional mobile noise and strongly enhances the desired speech, thereby improving the performance of CI devices in a noisy environment.

**Methods:**

The spectrum estimation and the array beamforming methods were combined to suppress the ambient noise. The directivity coefficient was estimated in the noise-only intervals, and was updated to fit for the mobile noise.

**Results:**

The proposed algorithm was realized in the CI speech strategy. For actual parameters, we use Maxflat filter to obtain fractional sampling points and cepstrum method to differentiate the desired speech frame and the noise frame. The broadband adjustment coefficients were added to compensate the energy loss in the low frequency band.

**Discussions:**

The approximation of the directivity coefficient is tested and the errors are discussed. We also analyze the algorithm constraint for noise estimation and distortion in CI processing. The performance of the proposed algorithm is analyzed and further be compared with other prevalent methods.

**Conclusions:**

The hardware platform was constructed for the experiments. The speech-enhancement results showed that our algorithm can suppresses the non-stationary noise with high SNR. Excellent performance of the proposed algorithm was obtained in the speech enhancement experiments and mobile testing. And signal distortion results indicate that this algorithm is robust with high SNR improvement and low speech distortion.

## Background

The clinical cochlear implant (CI) has good speech recognition under quiet conditions, but noticeably poor recognition under noisy conditions [1]. For 50% sentence understanding [2,3], the required signal to noise ratio (SNR) is between 5 and 15 dB for CI recipients, but only −10 dB for normal listeners. The SNR in the typical daily environment is about 5–10 dB, which results in <50% sentence recognition for CI users in a normal noise environment.

Most previous studies on recognition improvement have focused on the coding strategy, design of the electrode array, and stimulation adjustment of pitch recognition, as well as on the virtual electrode technique [4,5] and optical CIs [6]. More recent efforts have focused on the microphone array technique [7,8]. This array beamforming method promises to be more effective for situations in which the desired voice and ambient noise originate from different directions, the usual work environment for CI devices.

Speech-enhancement methods include single- and multichannel techniques. Spectral estimation methods are the most widely used single-channel techniques. Typical single-channel approaches, such as the spectral subtraction [9,10], Wiener filtering [11], and subspace approach [12], are based on estimations of the power spectrum or higher- order spectrum, assume the noise to be stationary, and use the noise spectrum in the nonspeech frame to estimate the speech-frame noise spectrum. Algorithm performance sharply weakens when the noise is non-stationary, or under typical situations with music or ambient speech noise.

The microphone array technique considers the signal orientation information and focuses on directional speech enhancement. Specifically, the generalized sidelobe canceller [13] and delay beamforming [14,15] use multiple microphones to record signals for spatial filtering. For CI devices, the generalized sidelobe canceller is overly complicated and requires too many microphones, conditions that exceed the capabilities of current CI devices. Delay beamforming technologies, such as the first-order differential microphone (FDM) [16] and adaptive null-forming method (ANF) [17,18], are adopted in hearing aids. These methods need only 2 microphones, which is an appropriate set-up for the CI size constraint and real-time processing.

CI devices are similar with the hearing aids in size constraint and the requirement of front-end noise suppression. So, for CI speech enhancement, one simple solution for CI speech enhancement is to directly utilize the microphone-array–based noise-reduction methods from the present hearing aids, in which the sensor-array techniques have been more widely used. However, the difference between CI devices and hearing aids is prominent, and a direct application of these algorithms to CI speech processing is not appropriate. Firstly, the principle is very different. CI devices transfer the acoustic signal to electrical stimulation into the cochlea wirelessly, and then the electrical pulses are used to directly stimulate the acoustic nerve to yield the auditory perception. But the hearing aids only need to change the corresponding gains in different subbands for multi-frequency signal loss. In brief, the hearing aid is only an amplifier with adjustable gain in different frequency band. Secondly, the application of the microphone array technique is different. Many algorithms for speech application were borrowed from the narrowband methods in radar and antenna. Algorithms for front-end enhancement are indispensable to match the CI speech strategy. Thirdly, the solution for low frequency roll-off may be different. The hearing aids need to calibrate and preset the subband gain based on user’s hearing loss. Therefore, in the hearing aid, one solution is to directly preset the subband gains in the filter banks in the processor by both taking the hearing loss and signal loss in microphone array algorithm into account. However, for CI devices with the modulated electrical pulse directly stimulate the cochlear nerves, we only need to adjust the algorithm loss. Finally, the signal distortion is different. When the enhanced signal is modulated by the CI speech strategy, the signal distortion will noticeably decreased (detailed analysis was given in the result section). Therefore, an array for a cochlear implant is similar to a hearing aid in the speech-enhancement situation, but is different for the actual algorithm design, such as the tradeoff between speech distortion and noise suppression.

The Frost algorithm [19,20], multiple input/output inverse method (MINT) [21], minimum-variance distortionless-response technique (MVDR) [22,23] and the binaural frequency-domain minimum-variance algorithm [24] are proposed presently, with excellent performance in some specific situations. Kates [25] used a novel five-microphone end-fire array, with an MVDR included, to construct an adaptive frequency-domain noise-reduction algorithm with higher SNR improved. However, this algorithm is overly complicated, and the five-microphone array also exceeds the CI size constraint.

In daily environment, we previously proposed a low-complexity beamforming with optimal parameter to suppress the environmental stationary noise [26]. But for the music and speech noise, we need a higher SNR to weaken these ambient noises, aiming to obtain more than 10 dB SNR for the CI front-end signal acquisition. This paper focuses on directional noise suppression with one directional ambient interference for CI devices. In typical situations in which CI users want to talk with a nearby person in a conference hall or a theater, the directional voice from the lecturer or film screen must be suppressed. To weaken the directional noise in such situations, a dual-channel CI speech-enhancement algorithm was introduced that combines the single-channel power spectrum estimation and the first-order differential microphone technique of the microphone array, for beamforming and noise prediction. Our algorithm uses the dual-channel power spectrums in the noise-only intervals, including the nonstationary noise, to estimate and update the noise directivity coefficient. For noise changing in normal human walking velocity, the proposed algorithm can avoid the noise leakage and is robust to mobile noise. For spectrum estimation based speech enhancement, the speech distortion is also unavoidable in our algorithm. But when the signal is modulated in the CI speech strategy, the speech distortion will sharply decrease and the speech quality noticeably improves. The experimental results indicate that the proposed algorithm successfully achieves the desired speech reconstruction and enhancement.

## Methods

**Figure 1:**
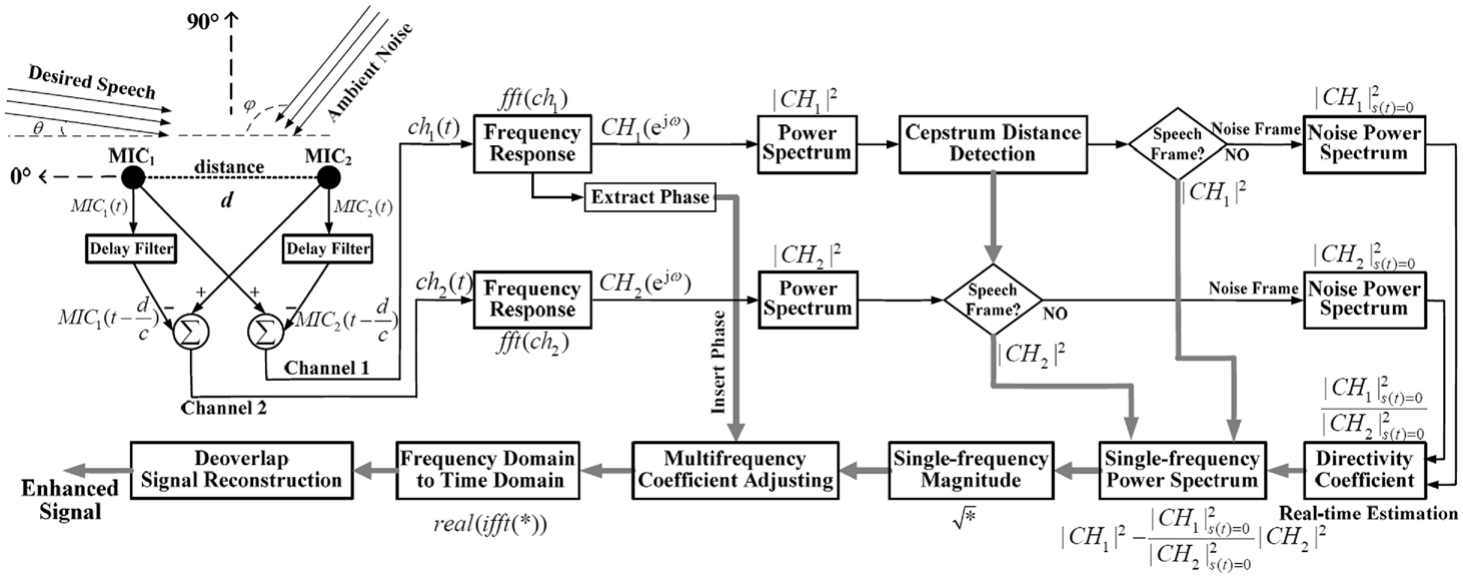
Dual-channel speech-enhancement algorithm based on real-time spectrum estimation

For the actual daily usage of CI devices, the front speech is the desired signal that needs to be enhanced, as shown in Figure 1 (signal azimuth θ approaches 0°). The noise, including ambient music and other speech signals, originates from another direction (azimuth *φ*).

Figure 1 shows the flow chart of the proposed dual-channel speech-enhancement algorithm. Signals recorded by two omnidirectional microphones and two delaying signals by the delay filters are summed to yield dual-channel outputs. Firstly, the signal frequency response in each channel is extracted to obtain the power spectrum and the cepstrum distance. The cepstrum distance differentiates the desired-speech and noise-only power spectrums. Then the noise-only power spectrums are adopted to estimate the noise directivity coefficient. The narrowband signal magnitude is estimated by these power spectrums, including the desired-speech and noise-only segments, and the directivity coefficient. Furthermore, the narrowband signal magnitude, also named as single-frequency magnitude, is adjusted to yield the multifrequency magnitude for the desired speech (broadband signal with the compensation for low-frequency loss). And the phase information in channel 1 is extracted and inserted for signal reconstruction to obtain the enhanced speech signal. Our proposed algorithm is theoretically depicted as below:

Two omnidirectional microphones are spaced a distance d apart. The desired speech comes from a direction *θ* (*θ*→ 0°) and the ambient noise from *φ*, both being recorded by MIC_1_ and MIC_2_. If we denote the desired speech and ambient noise obtained by MIC_1_ as s(t) and n(t), then the recorded signal by MIC_1_ can be written as

(1) $$MIC_{1}\left(t\right)=s\left(t\right)+n\left(t\right)$$

The spatial difference between the microphones results in a time delay for the recorded signal by MIC_2_, shown as

(2) $$MIC_{2}\left(t\right)=s\left(t-d/c\cdot \cos\theta \right)+n\left(t-d/c\cdot \cos\varphi \right)$$

where c is the speed of the sound. The recorded signals MIC_1_(t) and MIC_2_(t) are both delayed by the fractional delay filter for a fixed time d/c. Based on the FDM method, ch_1_(t) and ch_2_(t) can be defined as the sum of the original recorded signals and the corresponding delayed signals, described by Eqs. (3) and (4):

(3) $$ch_{1}\left(t\right)=s\left(t\right)+n\left(t\right)-s\left(t-\frac{d}{c}-\frac{d}{c}\cos\theta \right)-n\left(t-\frac{d}{c}-\frac{d}{c}\cos\varphi \right)$$

(4) $$ch_{2}\left(t\right)=s\left(t-\frac{d}{c}\cos\theta \right)+n\left(t-\frac{d}{c}\cos\varphi \right)-s\left(t-\frac{d}{c}\right)-n\left(t-\frac{d}{c}\right)$$

The frequency responses for time-domains ch_1_(t) and ch_2_(t) can be expressed as

(5) $$CH_{1}\left(e^{\mathrm{j\omega}}\right)=\left(1-e^{-j\omega d/c\cdot \left(1+\cos\theta \right)}\right)S\left(e^{\mathrm{j\omega}}\right)+\left(1-e^{-j\omega d/c\cdot \left(1+\cos\varphi \right)}\right)N\left(e^{\mathrm{j\omega}}\right)$$

(6) $$CH_{2}\left(e^{\mathrm{j\omega}}\right)=\left(e^{-j\omega d/c\cdot \cos\theta }-e^{-j\omega d/c}\right)S\left(e^{\mathrm{j\omega}}\right)+\left(e^{-j\omega d/c\cdot \cos\varphi }-e^{-j\omega d/c}\right)N\left(e^{\mathrm{j\omega}}\right)$$

where *ω* = 2*πf*, f corresponds to a narrowband signal frequency. And the corresponding power spectrum for ch_1_(t) is:

(7) $${\left|CH_{1}\left(e^{\mathrm{j\omega}}\right)\right|}^{2}=2\left(1-\cos\left(\omega d/c\cdot \left(1+\cos\theta \right)\right)\right){\left|S\left(e^{\mathrm{j\omega}}\right)\right|}^{2}+A_{1}A_{2}^{*}\left(S\left(e^{\mathrm{j\omega}}\right)N^{*}\left(e^{\mathrm{j\omega}}\right)\right)+A_{1}^{*}A_{2}\left(S^{*}\left(e^{\mathrm{j\omega}}\right)N\left(e^{\mathrm{j\omega}}\right)\right)+2\left(1-\cos\left(\omega d/c\cdot \left(1+\cos\varphi \right)\right)\right){\left|N\left(e^{\mathrm{j\omega}}\right)\right|}^{2}$$

where $A_{1}=1-e^{-j\omega \frac{d}{c}\left(1+\cos\theta \right)}$ and $A_{2}=1-e^{-j\omega \frac{d}{c}\left(1+\cos\varphi \right)}$.

In Eq. (7), each framed data set (of about 23 ms in duration) is used to calculate the corresponding statistical average of the power spectrum. For daily CI application, the desired signal s(t) and ambient noise n(t) are uncorrelated, given in Eq. (8).

(8) $$E\left(S\left(e^{\mathrm{j\omega}}\right)N^{*}\left(e^{\mathrm{j\omega}}\right)\right)=E\left(S^{*}\left(e^{\mathrm{j\omega}}\right)N\left(e^{\mathrm{j\omega}}\right)\right)=0$$

Substituting Eq. (8) into Eq. (7) gives the statistical power spectrum set in channel 1:

(9) $$E\left({\left|CH_{1}\left(e^{\mathrm{j\omega}}\right)\right|}^{2}\right)=2\left(1-\cos\left(\omega d/c\left(1+\cos\theta \right)\right)\right)E\left({\left|S\left(e^{\mathrm{j\omega}}\right)\right|}^{2}\right)+2\left(1-\cos\left(\omega d/c\left(1+\cos\varphi \right)\right)\right)E\left({\left|N\left(e^{\mathrm{j\omega}}\right)\right|}^{2}\right)$$

For CI devices, the desired speech generates from the front, and the signal direction *θ* approaches 0°. Thus, cos *θ* ≈ 1, which simplifies Eq. (9) and gives

(10) $$E\left({\left|CH_{1}\left(e^{\mathrm{j\omega}}\right)\right|}^{2}\right)=2\left(1-\cos\left(2\omega d/c\right)\right)E\left({\left|S\left(e^{\mathrm{j\omega}}\right)\right|}^{2}\right)+2\left(1-\cos\left(\omega d/c\cdot \left(1+\cos\varphi \right)\right)\right)E\left({\left|N\left(e^{\mathrm{j\omega}}\right)\right|}^{2}\right)$$

Similarly, the simplified statistical power spectrum for each framed data in channel 2 is written as:

(11) $$E\left({\left|CH_{2}\left(e^{\mathrm{j\omega}}\right)\right|}^{2}\right)=2\left(1-\cos\left(\omega d/c\left(1-\cos\varphi \right)\right)\right)E\left({\left|N\left(e^{\mathrm{j\omega}}\right)\right|}^{2}\right)$$

Seen from Eq. (10), the power spectrum for each framed data set in channel 1 includes the power spectra of desired speech and the ambient noise. Equation (11) only contains the power spectrum of the ambient noise in channel 2. In addition, the power spectra of the noise in channels 1 and 2 are different, which are functions of the noise azimuth.

To estimate the directivity of the ambient noise, the power spectra of the 2 channels are calculated at the noise frame of desired speech, where s(t) = 0 and $S\left(e^{\mathrm{j\omega}}\right)$=0:

(12) $$E\left({\left|CH_{1}\left(e^{\mathrm{j\omega}}\right)\right|}^{2}\right){|}_{s\left(t\right)=0}=2\left(1-\cos\left(\omega d/c\cdot \left(1+\cos\varphi \right)\right)\right)E\left({\left|N\left(e^{\mathrm{j\omega}}\right)\right|}^{2}\right){|}_{s\left(t\right)=0}$$

(13) $$E\left({\left|CH_{2}\left(e^{\mathrm{j\omega}}\right)\right|}^{2}\right){|}_{s\left(t\right)=0}=2\left(1-\cos\left(\omega d/c\cdot \left(1-\cos\varphi \right)\right)\right)E\left({\left|N\left(e^{\mathrm{j\omega}}\right)\right|}^{2}\right){|}_{s\left(t\right)=0}$$

For each framed data set, the statistical average of the power spectrum can be used to estimate the power spectrum for desired speech, and yield the magnitude estimation in Eq. (14).

(14) $$\left|\hat{S}\left(e^{\mathrm{j\omega}}\right)\right|=\frac{{\left|E\left({\left|CH_{1}\left(e^{\mathrm{j\omega}}\right)\right|}^{2}\right)-\frac{E\left({\left|CH_{1}\left(e^{\mathrm{j\omega}}\right)\right|}^{2}\right){|}_{s\left(t\right)=0}}{E\left({\left|CH_{2}\left(e^{\mathrm{j\omega}}\right)\right|}^{2}\right){|}_{s\left(t\right)=0}}E\left({\left|CH_{2}\left(e^{\mathrm{j\omega}}\right)\right|}^{2}\right)\right|}^{0.5}}{2\sin\left(2\pi fd/c\right)}$$

Eq. (14) indicates the algorithm for magnitude estimation must obtain the power spectrum of the framed data set of each channel, as well as the power spectrum of each channel at the noise-only frame.

$E\left({\left|CH_{1}\left(e^{\mathrm{j\omega}}\right)\right|}^{2}\right){|}_{s\left(t\right)=0}/E\left({\left|CH_{2}\left(e^{\mathrm{j\omega}}\right)\right|}^{2}\right){|}_{s\left(t\right)=0}$, defined as the directivity coefficient, is a function of the noise azimuth *φ*. The noise power spectrums in channels 1 and 2 are different, which are also the functions of the noise azimuth. The directivity coefficient is used to estimate the gain of noise power spectrums between channels 1 and 2. When the inter-channel noise gain is estimated accurately and the two channels’ noise power spectrums are then balanced to approximately the same, the ambient noise can be attenuated and the desired speech signal can be extracted. The noise directivity coefficient is further analyzed and simplified by Eq. (15):

(15) $$\frac{E\left({\left|CH_{1}\left(e^{\mathrm{j\omega}}\right)\right|}^{2}\right){|}_{s\left(t\right)=0}}{E\left({\left|CH_{2}\left(e^{\mathrm{j\omega}}\right)\right|}^{2}\right){|}_{s\left(t\right)=0}}=\frac{\sin^{2}\left(0.5\omega d/c\cdot \left(1+\cos\varphi \right)\right)}{\sin^{2}\left(0.5\omega d/c\cdot \left(1-\cos\varphi \right)\right)}\approx \frac{{\left(0.5\omega d/c\cdot \left(1+\cos\varphi \right)\right)}^{2}}{{\left(0.5\omega d/c\cdot \left(1-\cos\varphi \right)\right)}^{2}}=\cot^{4}\left(\varphi /2\right)$$

For an actual CI size constraint of d≈ 0.01 m, the directivity coefficient approaches to cot ^4^(*φ*/2). This result indicates that the estimation of the directivity coefficient is robust, because it only depends on the noise direction *φ*. This simplified form of noise directivity coefficient also implies that, for noise direction with slowly varying, the adjusted gain for noise reduction can be accurately obtained with excellent algorithm stability.

### Realization

#### Fractional delay

In Figure 1, the recorded signals MIC_1_(t) and MIC_2_(t) are sampled at the 44.1 kHz sampling rate by the AD converter as MIC_1_(n) and MIC_2_(n). These digital signals are then delayed by the fractional delay filter with an algorithm offset of d/c. In our hardware platform, the system design specifies the intermicrophone distance d to be at or near 1 cm, corresponding to 1.297 sampling points.

To obtain this accurate fractional delaying, we use the maximal flat (Maxflat) criteria [27-30] to design a fourth-order finite impulse response (FIR) filter for the required system delaying. For CI devices, the required speech energy is primarily in the low-frequency band, peaking near 1 kHz; this Maxflat FIR filter matches the desired speech characteristic well. And the fourth-order Maxflat FIR filter is given by

(16) $$h\left(n\right)=\left[-0.0400,0.6995,0.4433,-0.1220,0.0192\right]$$

For f_s_ = 44.1 kHz, the digital signals MIC_1_(n) and MIC_2_(n) are delayed, with d/c offsets of MIC_1_(n-1.297) and MIC_2_(n-1.297), respectively.

**Figure 2:**
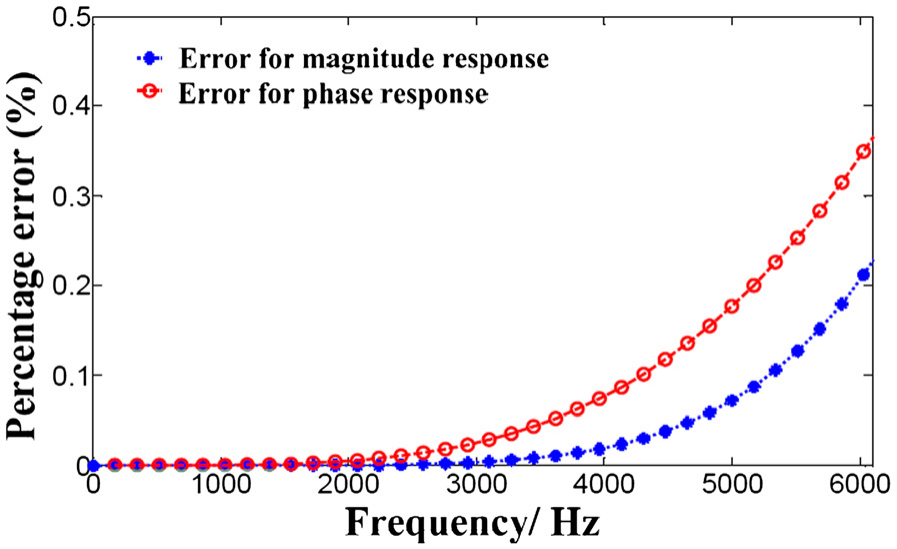
**Errors of magnitude and phase responses of the Maxflat FIR filter and the ideal filter at 0–6000 Hz**.

For the time-domain delay of 1.297 sampling points, the ideal system frequency response is $e^{-j\omega \times 1.297}$, which is a linear-phase all-pass filter. The proposed fourth-order Maxflat FIR filter approaches the ideal filter at the range of 0–6000 Hz. The errors of magnitude and phase responses are plotted in Figure 2.

The proposed fourth-order Maxflat FIR filter agrees well with the ideal filter at the signal range of 0–6000 Hz, a range that includes most of the subbands of the CI filter bank. The maximal error for the magnitude response and phase response are less than 0.3% and 0.4%, respectively. Additionally, this filter can obtain the required delaying signal easily, with low computation complexity.

This Maxflat digital filter is used to cover frequencies between 0 Hz and 6000 Hz as the CIS strategy does. The range of the CIS actually depends on the corner and center frequencies of the filters, therefore, the frequency range will change based on different channel quantities. But both the present CI filter banks (8, 16, 24 channels etc.) are primarily within these range. So, the required signal delaying is accurate.

### Directivity estimation and noise-frame identification

The directivity coefficient (Figure 1 and Eq. (15)) is obtained from the power spectrums of the 2 channels at the noise-only frames, which correspond to the time-domain signals of ch_1_(t)|_s(t)=0_ and ch_2_(t)|_s(t)=0_, respectively. We used the cepstrum [31-33] method to differentiate the desired speech frame and the noise frame.

The anterior several frames of data are considered to be noise. The cepstrum vector is denoted as C, which is expressed by a series of vector coefficients c_i_. Then, the spectrum density function is given by Eq. (17)

(17) $$logS\left(\omega \right)=\sum_{n=-\infty }^{\infty }c_{n}e^{-j\omega n}\;\text{and}\;c_{0}=\int_{-\pi }^{\pi }logS\left(\omega \right)\frac{d\omega }{2\pi }$$

The average cepstrum coefficients of the anterior several frames are used to estimate and obtain the cepstrum vector C. The cepstrum vector is updated by the current cepstrum vector C_i_ and the previous cepstrum vector C_i-1_. The update equation is *C*_*i*_ = *βC*_*i*_ + (1 − *β*)*C*_*i*−1_, where *β* is the update weight. In our algorithm, *β* is 0.85 and the corresponding cepstrum distance is given by

(18) $$d_{\mathrm{cep}}{|}_{\left(i+1\right)}=A\sqrt{{\left(c_{0}{|}_{\left(i+1\right)}-c_{0}{|}_{i}\right)}^{2}+2\sum_{n=1}^{p}{\left(c_{n}{|}_{\left(i+1\right)}-c_{n}{|}_{i}\right)}^{2}}$$

Equation (18) with a predefined threshold value is used to differentiate the noise frames for both channels, ch_1_(t)|_s(t)=0_ and ch_2_(t)|_s(t)=0_. Then, the corresponding power spectra are used to obtain the real-time noise directivity coefficient.

### Broadband signal adjustment

The speech is a broadband signal with multifrequency information. For the first order differential microphone, we previously proposed the normalized beamforming method for the gain [34] adjustment. But in this paper, we can compensate the gains directly in the dual-channel algorithm. Equation (14) provides the magnitude estimation for the desired speech changes as a function of signal frequency f. The coefficient is a function of the signal frequency denoted as Eq. (19).

(19) $$\lambda \left(f\right)=1/\left(2\sin\left(2\pi fd/c\right)\right)$$

Therefore, the magnitude estimation for the desired speech is modulated by the *λ*(*f*) to change its multifrequency gain. This coefficient function is monotone, decreasing between 100 and 6000 Hz. We design a digital filter to approximate *λ*(*f*). The codomain of *λ*(*f*) is between 0.6 and 27 when the frequency is between 100 and 6000 Hz. Because the maximal magnitude response of a filter is always 1, the desired coefficient function is actually written as Eq. (20).

(20) $$\lambda^{\prime }\left(f\right)=\frac{1}{30}\lambda \left(f\right)=\frac{1}{60\sin\left(2\pi fd/c\right)}$$

**Figure 3:**
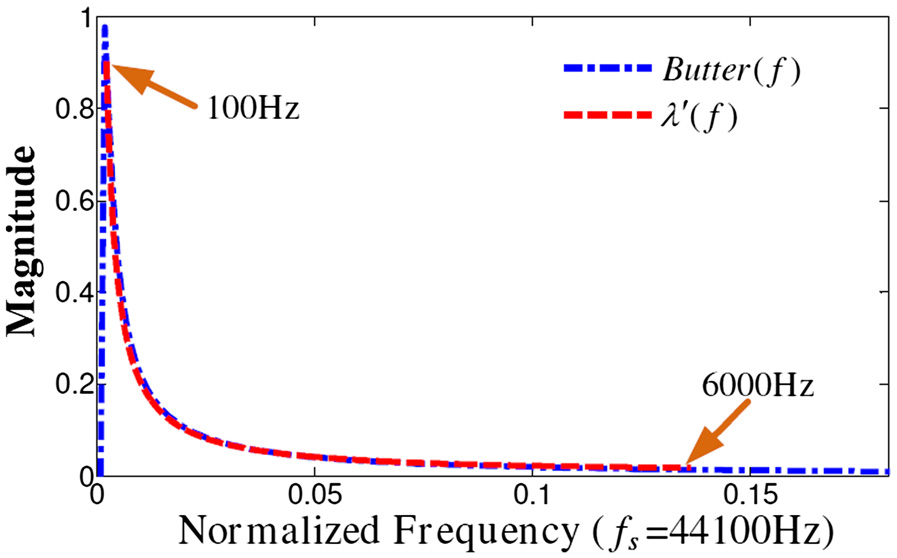
**Comparison of the Butterworth filter Butter(f) and the desired coefficient function*λ*’(*f*)**.

We design a 1^st^ order Butterworth filter, *Butter*(*f*), with band-pass cutoff frequencies of 0.00045 f_s_ and 0.0045 f_s_, to approach the coefficient function *λ*’(*f*), as shown in Figure 3.

The proposed filter for multi-frequency adjusting is highly consistent with the required coefficient function between 100 and 6000 Hz. Because *λ*(*f*) = 30*λ*’(*f*), the filtered signal needs an additional 30 times gain (or 29.54 dB) for signal energy rebalancing.

**Figure 4:**
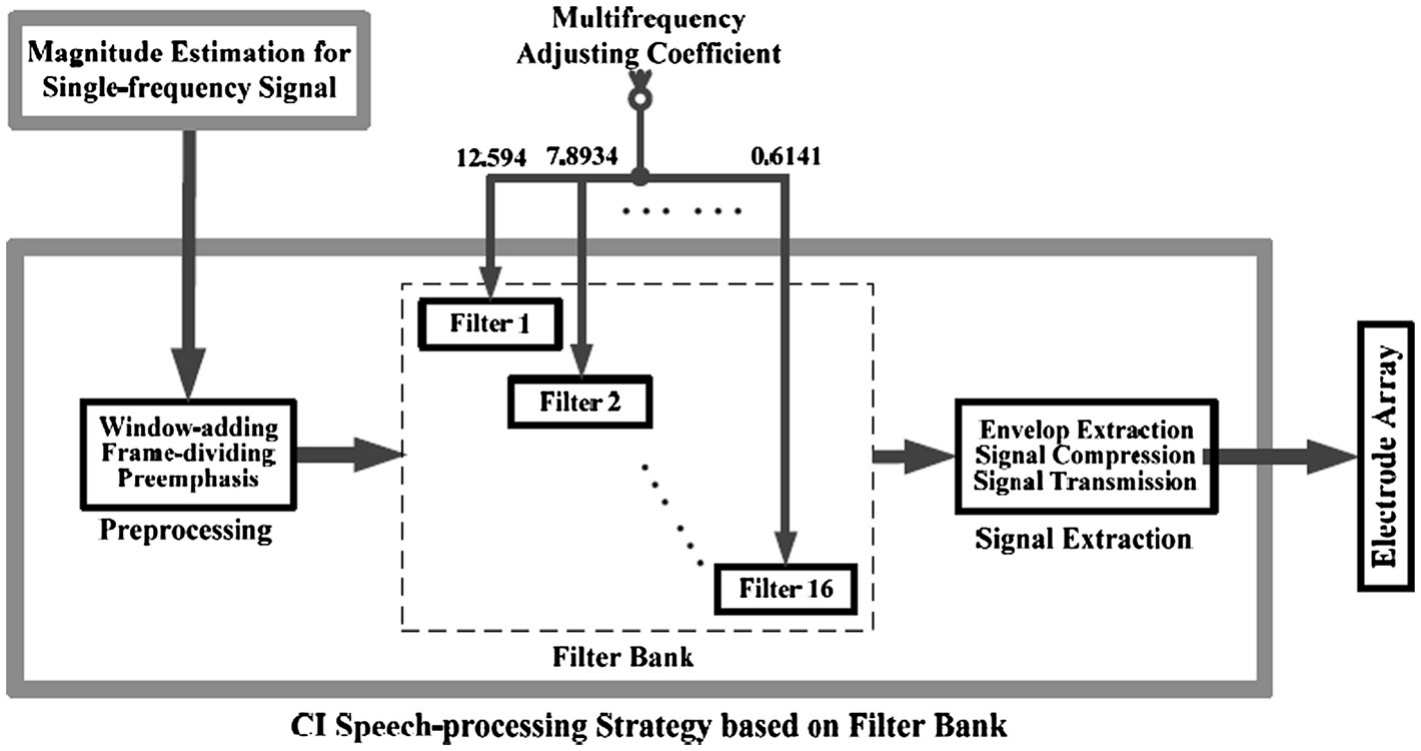
Transfer of the adjusting coefficients to the CI speech processor for the speech strategy based on the filter bank.

For a CI speech-processing strategy based on a filter bank, such as the continuous interleaved sampling strategy (CIS [35]) or advanced combined encoder strategy (ACE [36]), the adjusting coefficients for the multi-frequency signal can be transferred directly to the corresponding subband filters. According to the characteristics of the cochlea [37], the speech signal can be divided into 16 bands (Table 1) and the corresponding band center frequencies are also listed in Table 1. Eq. (19) is used to calculate the desired adjusting coefficient for each band of the filter bank, shown in this Table and Figure 4.

**Table 1:** Parameters of each sub-band in CI filter bank and the corresponding adjusting coefficients

	**Band edge (Hz)**	**Center frequency (Hz)**	**Adjusting coefficient**
Channel 1	[156, 276]	216	12.5294
Channel 2	[276, 410]	343	7.8934
Channel 3	[410, 560]	485	5.5861
Channel 4	[560, 730]	645	4.2047
Channel 5	[730, 922]	826	3.2883
Channel 6	[922, 1138]	1030	2.6428
Channel 7	[1138, 1380]	1259	2.1685
Channel 8	[1380, 1653]	1517	1.8071
Channel 9	[1653, 1960]	1807	1.5255
Channel 10	[1960, 2305]	2133	1.2135
Channel 11	[2305, 2694]	2500	1.1217
Channel 12	[2694, 3131]	2913	0.9752
Channel 13	[3131, 3623]	3377	0.8557
Channel 14	[3623, 4176]	3840	0.7578
Channel 15	[4176, 4798]	4487	0.6781
Channel 16	[4798, 5498]	5148	0.6141

Figure 4 describes the transmission of multi-frequency adjusting coefficients to the CI processor. This method of directly transmitting the parameters to the CI filter bank requires very little additional calculation, which is suitable to the situation of a filter bank–based strategy. For the situation in which the CI processor uses the speech-processing strategy without a filter bank, the proposed Butterworth filter (Figure 3) should be adopted for the coefficient adjusting.

### Signal reconstruction

In our CI speech-enhancement platform, the sampling rate is 44.1 kHz and the Hamming window is used for framing, with a window length of 1024 sampling points. Each frame is about 23 ms in duration, with 50% overlap.

The speech magnitude is estimated by Eq. (14). The signal phase of the original data (channel 1) is used directly for signal construction, because the human cochlea is relatively insensitive to phase information. The gain of the single-frequency signal is adjusted by the proposed Butterworth filter or by directly transmitting the adjusting coefficients in the filter-bank based CI processor. This broadband signal, expressed in the form of frequency response, is processed by the subsequent processing of the inverse Fourier transform and deoverlapping to reconstruct the enhanced speech signal.

## Results

### Hardware platform

**Figure 5:**
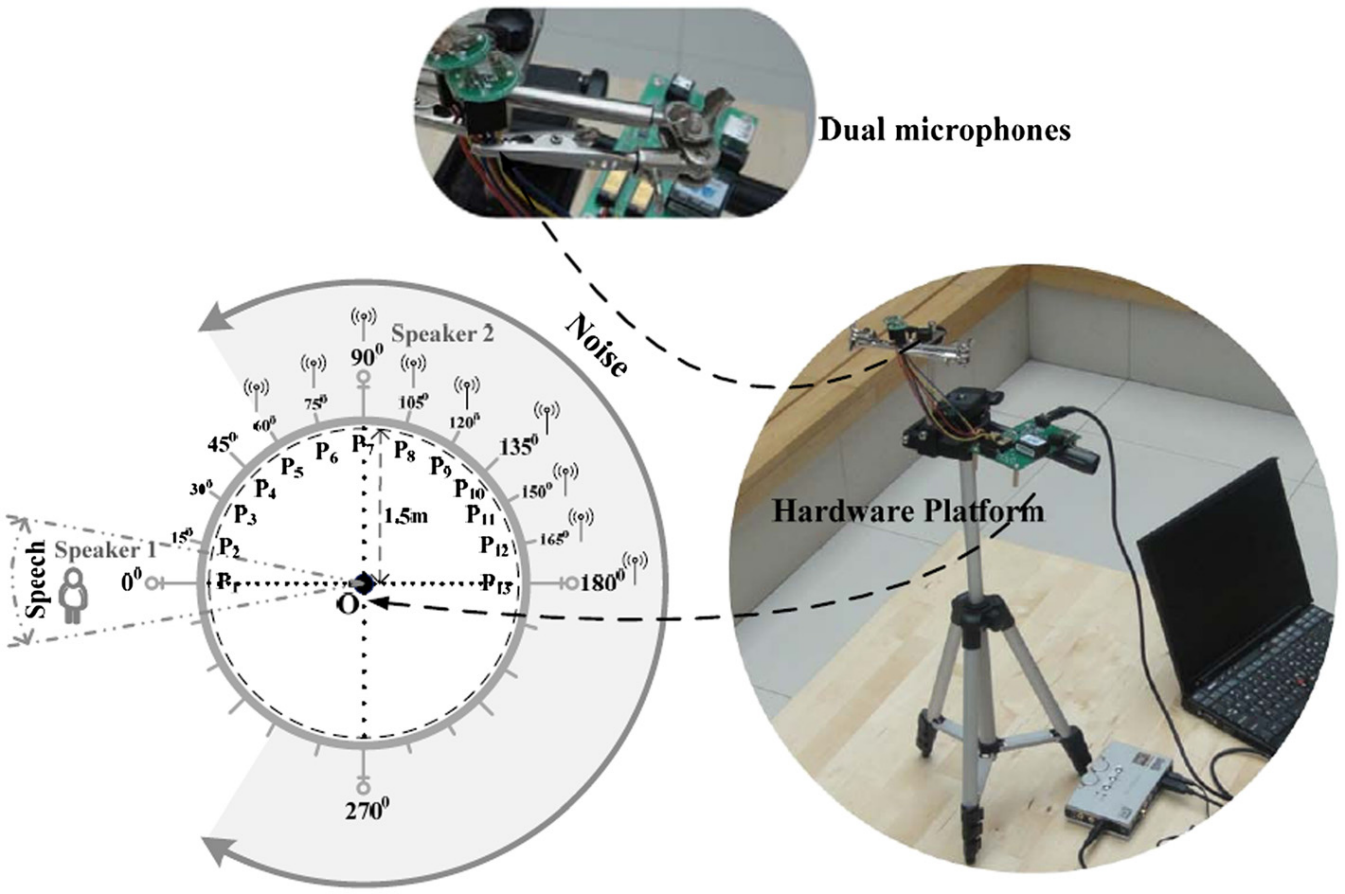
Dual-channel CI front-end hardware platform.

A dual-channel CI front-end hardware platform was constructed (Figure 5). The dual microphones were linearly spaced 1 cm apart. The recorded signals, which were obtained with a real-time acquisition process controlled by a software interface, were transmitted to the computer.

The experiments were carried out in a chamber, or to be extract, an actual office measured by 8 m× 8 m× 4 m with the room reverberation time T_60_ = 450 ms. Two microphone modules, placed at the center (O), recorded the signal. P_1_-P_12_, which represent 12 testing points at 15° intervals, were marked 1.5 m from the microphones arranged in a semicircle. P_1_ indicates the forward direction for playing the desired speech by Speaker 1; the other locations (P_2_-P_12_) indicate the directions for playing the ambient noise by Speaker 2. The recorded signals by this hardware system, after amplification, filtering, and analogue to digital conversion, were transmitted to the computer via a USB interface for further analysis.

### Speech-enhancement result

**Figure 6:**
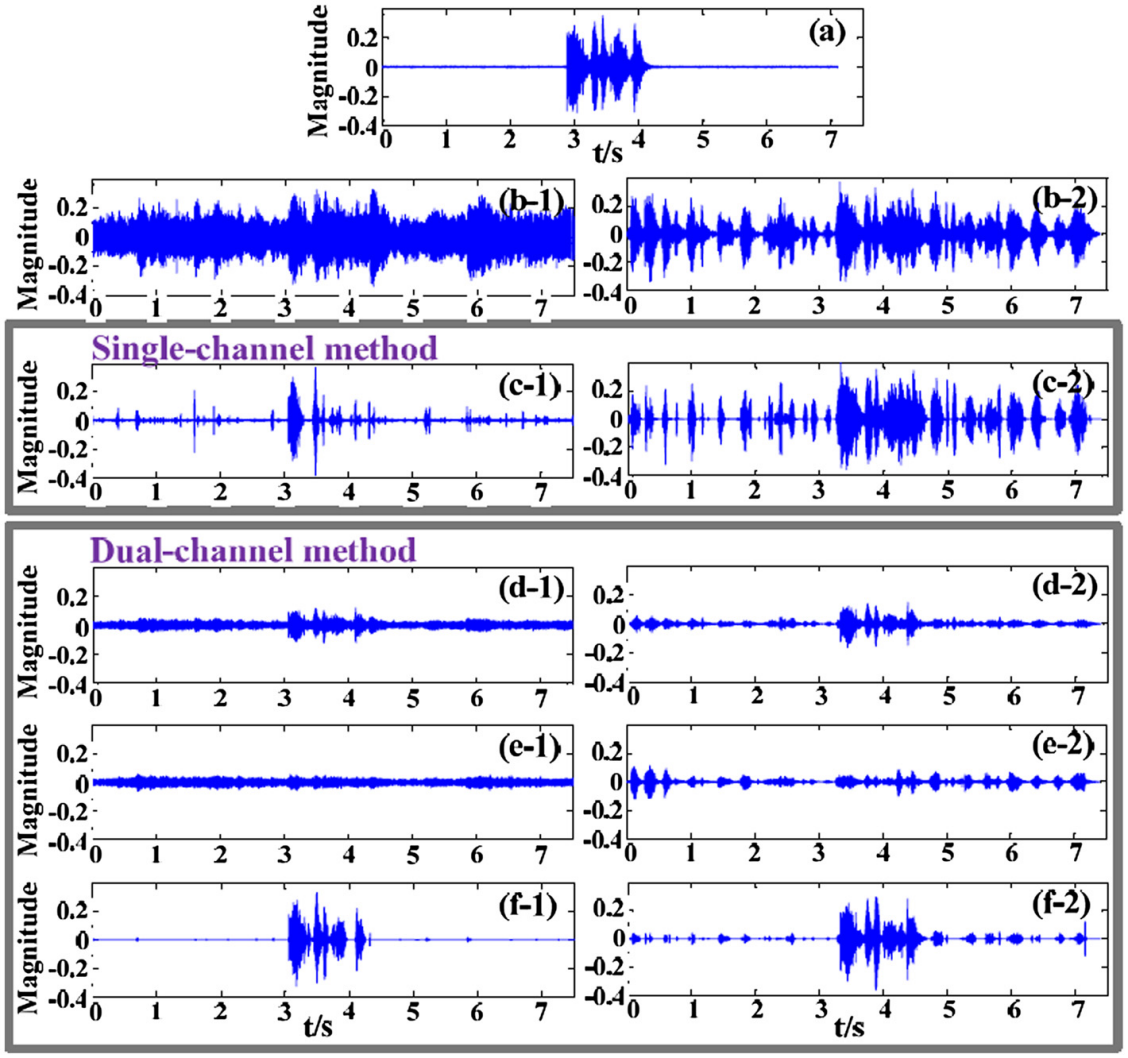
**Speech-enhancement results: comparison of the proposed algorithm and the single-channel method.** (**a**) Original signal played as the desired speech. (**b**) Signal recorded by the omnidirectional microphone at O (**b-1**: includes the desired speech and the music noise; **b-2**: includes the desired speech and the ambient speech noise). The corresponding speech-enhancement results for the single-channel method (**c-1** and **c-2**) and our proposed algorithm (**f-1** and **f-2**) are plotted, and **d-1**,**e-1** and **d-2**, **e-2** are the corresponding signal outputs of channel 1 and channel 2 for music and speech noises respectively.

For this test, a speech signal (i.e., the English sentence “I heard you called me” spoken by a native speaker (American English) as material) was played by Speaker 1 at P_1_. Speaker 2 played ambient noise, including 2 types of noise signals, at P_7_. One noise signal was the theme song “My Heart Will Go On” from the movie Titanic; the other was the speech signal in an interviewing scene, with part of the speech content being “My background and work experience are tailor-made for this position. I studied marketing as an undergrad here in Taiwan”. The desired speech and the ambient noise were played at the same power. For this situation, the SNR was approximately 0 dB, which is an extremely poor noise environment. The enhancement results were compared with those of the single-channel method of spectral subtraction (Figure 6).

Figure 6 (a) is the original waveform of the desired speech, played by Speaker 1 in P_1_. Speaker 2 located at P_7_ plays the noise. Figure 6 (b-1) and (b-2) are the plots recorded by the hardware platform (located at O) corresponding to the situations of music noise and speech noise, respectively.

In this paper, the improved SNR is defined by Eq. (21) [38,39]:

(21) $$\Delta SNR=\sum_{j=1}^{J}w_{j}\left(SNR_{j,out}-SNR_{j,in}\right)$$

where J is the frequency band quantity, *w*_*j*_ is the corresponding weight for different band given in [39], and the input SNR and output SNR is given by Eqs. (22) and (23).

(22) $$SNR_{\mathrm{in}}=10\mathrm{lg}\left(\frac{\sum_{n}{\left|s\left(n\right)\right|}^{2}}{\sum_{n}{\left|n\left(n\right)\right|}^{2}}\right)$$

(23) $$SNR_{\mathrm{out}}=10\mathrm{lg}\left(\frac{\sum_{n}{\left|\hat{s}\left(n\right)\right|}^{2}}{\sum_{n}{\left|\hat{n}\left(n\right)\right|}^{2}}\right)$$

where the output SNR uses the estimation of *ŝ*(*n*) and $\left|\hat{n}\left(n\right)\right|$ to obtain.

For the music noise, use of the single-channel method (panel c-1) based on spectral subtraction weakened most of the music noise, but much of the transient impulse at the nonstationary part of the music noise remained. Panels d-1 and e-1 plot the signal outputs in channel 1 and channel 2 in our dual-channel system. Comparison of (d-1) and (e-1), the magnitude attenuation or enhancement were different for the desired speech and music noise. And this characteristic can remarkably be seen in (d-2) and (e-2) for the ambient speech noise. The waveforms of the two channels were similar in time domain, but the gains in channel 2 were discrepant, with respectively about 0.3 and 2.8 gains for the desired speech and ambient speech compared with channel 1. These gains changed when the noise moved. The previous directivity coefficient in our algorithm was used to estimate the noise gain between channels 1 and 2 in the noise-only intervals. For accurate noise gain estimation, two channels’ noise power can be adjusted to nearly the same but noticeably discrepant for the power of the desired speech. Then the desired speech can be extracted from the subtraction of the adjusted signals in these two channels. Our proposed method suppressed the overall music noise, including the instantaneous noise (panel f-1). This method also suppressed the ambient speech noise, with nearly 20 dB SNR improvement (panel f-2). The single-channel method did not adequately suppress the ambient speech noise (panel c-2). The comparison indicates that the proposed dual-channel speech-enhancement algorithm successfully suppresses the nonstationary noise, which adds to its practical value.

**Figure 7:**
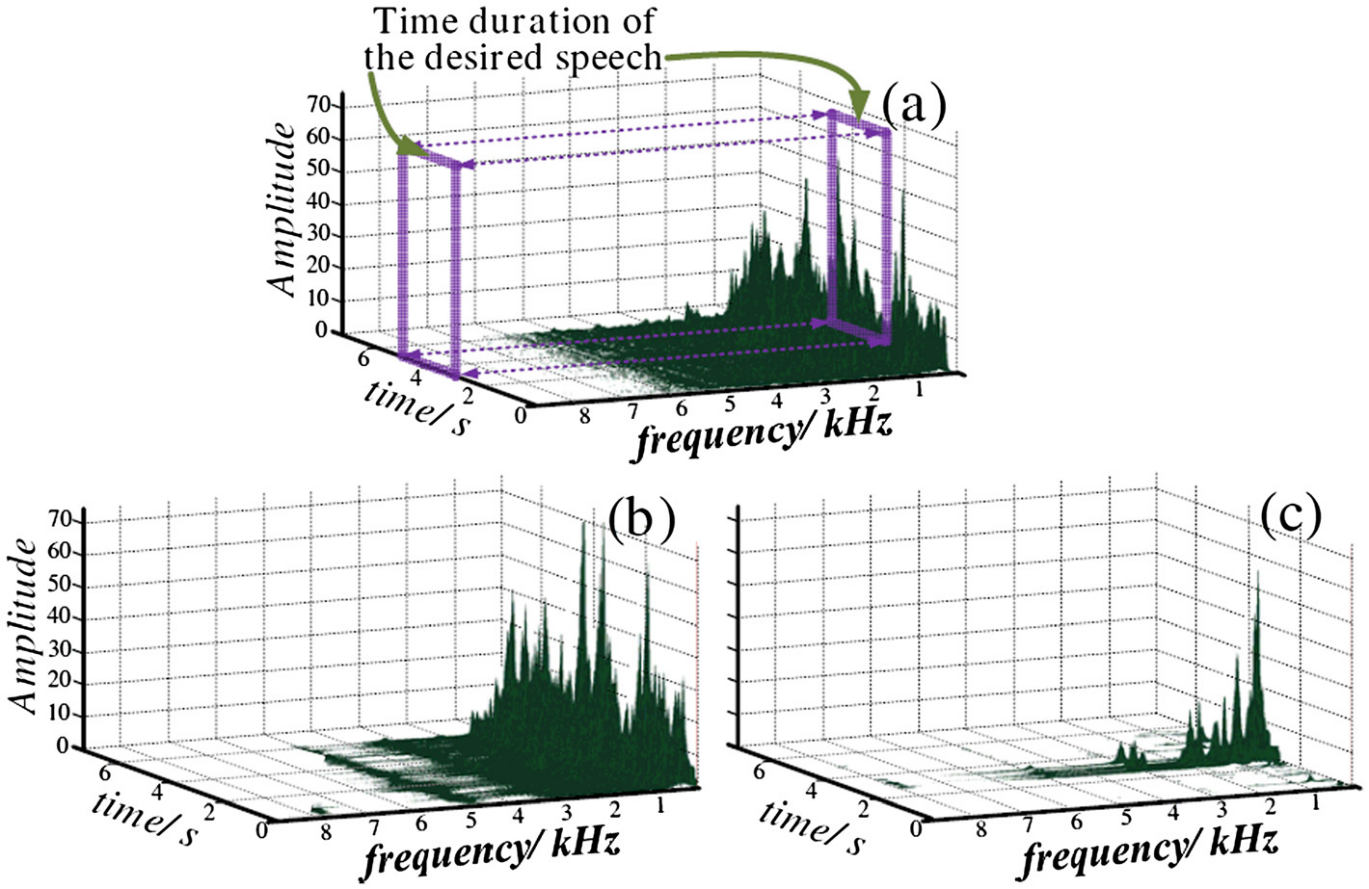
Comparison of the time-frequency energy distributions of the original signal (a), after modulation of the CIS strategy (b), and after enhancement by the proposed dual-channel algorithm and modulation of the CIS strategy (c).

The enhanced speech signal was further processed in CI speech processor. The CI speech strategy extracts and encodes the signal and then wirelessly transmits it to the electrode array. We adopted the widely used CIS strategy. The sine simulation model was used to implement the CI signal-processing. The energy distribution after CIS processing is shown in Figure 7.

For this test, the original signal, recorded by the platform, contained desired speech and ambient music noise. The signal duration was 7.5 sec and the desired speech was located in the time axis approximately between 3 sec and 5 sec. Figure 7 (a) describes the time-frequency energy distribution of original signal, which was dispersed in the frequency range between 0 and 6000 Hz.

Figure 7(b) describes the energy distribution of the signal after the modulation of the CIS strategy. This speech strategy divided the original signal by the filter bank and then modulated the subband signal, by using the center frequency of each band to characterize its corresponding information for further speech synthesis. The plot indicates that the CIS modulation only changed the frequency-domain energy distribution, but maintained the time-domain energy distribution. As a result, the signal energy concentrated in the corresponding center frequency of each subband, and the ambient noise in the time domain was not suppressed.

Figure 7(c) describes the energy distribution of the signal after enhancement by the proposed dual-channel algorithm and modulation of the CIS strategy. The energy distribution changed in the time domain, primarily between 3 sec and 5 sec, and the ambient noise was sharply weakened.

Together, the plots in Figure 7 indicate that the speech enhancement can achieve the following 2 purposes. First, the desired speech remained while the ambient noise was sharply suppressed, which improved the CI speech recognition. Second, the global signal energy was lowered, and the CI battery life was prolonged, because information from the signal and the energy both are transmitted wirelessly to the inner part of the CI device.

### Algorithm robustness and signal distortion analysis

**Figure 8:**
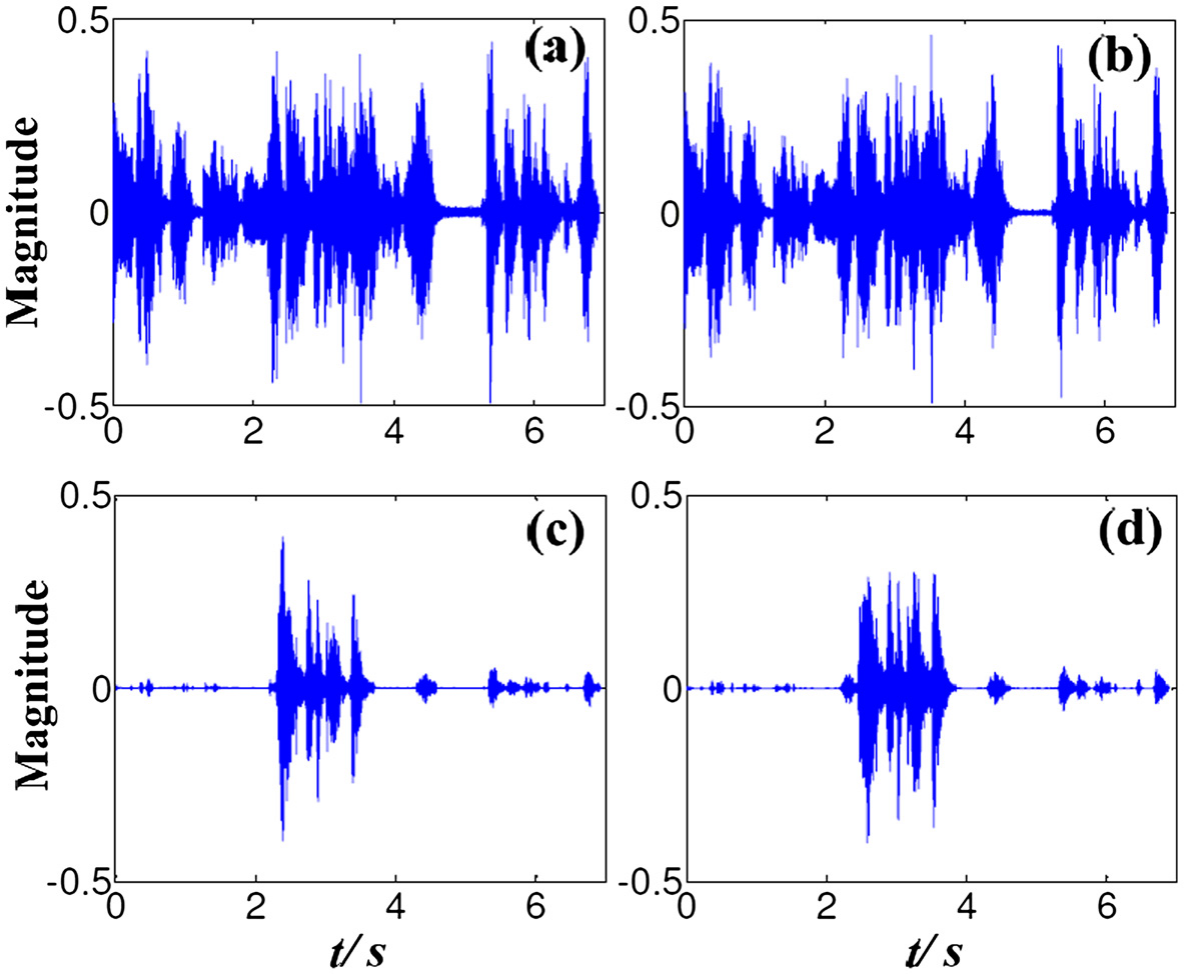
**Test of algorithm robustness for moving noise.** The original signals as the noise moves, based on a center position of 90° or 135°, are plotted in (**a**) and (**b**), respectively. The corresponding speech-enhancement results are plotted in (**c**) and (**d**), respectively.

The test aims to the analysis of algorithm robustness when the ambient noise was moving. The desired speech was played by Speaker 1 (P_1_ in Figure 5). Speaker 2 (located at P_7_, about 90° azimuth) played the ambient noise. During the testing, Speaker 2 moved back and forth at a speed of 1 m/s, corresponding to normal human walking velocity. The moving range was about 30°, from 75° to 105°. Another test, with the speaker located at P_10_ and moving from 120° to 150° at the same speed, was performed. The speech-enhancement results are shown in Figure 8.

For the tests in these 2 situations (moving noises from 90° and 135°), the original signals recorded by the omnidirectional microphone were plotted in (a) and (b), respectively. The original signal consists of the desired speech and the ambient speech noise. The noise suppression results are plotted in (c) and (d), respectively. A comparison of these plots reveals that the proposed algorithm also effectively weakens moving noise, with an SNR improvement of about 15 dB. The conventional noise-reduction methods need to reconvergent in algorithm for coefficients updating, and will always result in noise leakage and noticeable SNR decrease. The mentioned MVDR method, one of the most widely used adaptive beamformer, can choose and adjust the filter coefficients to minimize the output power with the constraint that the desired signal is not to be distorted. For moving noise, the MVDR method will also partly result in noise leakage, which attenuates the algorithm performance. The proposed algorithm calculates the noise directivity coefficient for moving noise, and also remains excellent performance with a few attenuation of SNR. As shown in result, the proposed algorithm is advantageous to avoid the noise leakage and is robust to mobile noise.

**Figure 9:**
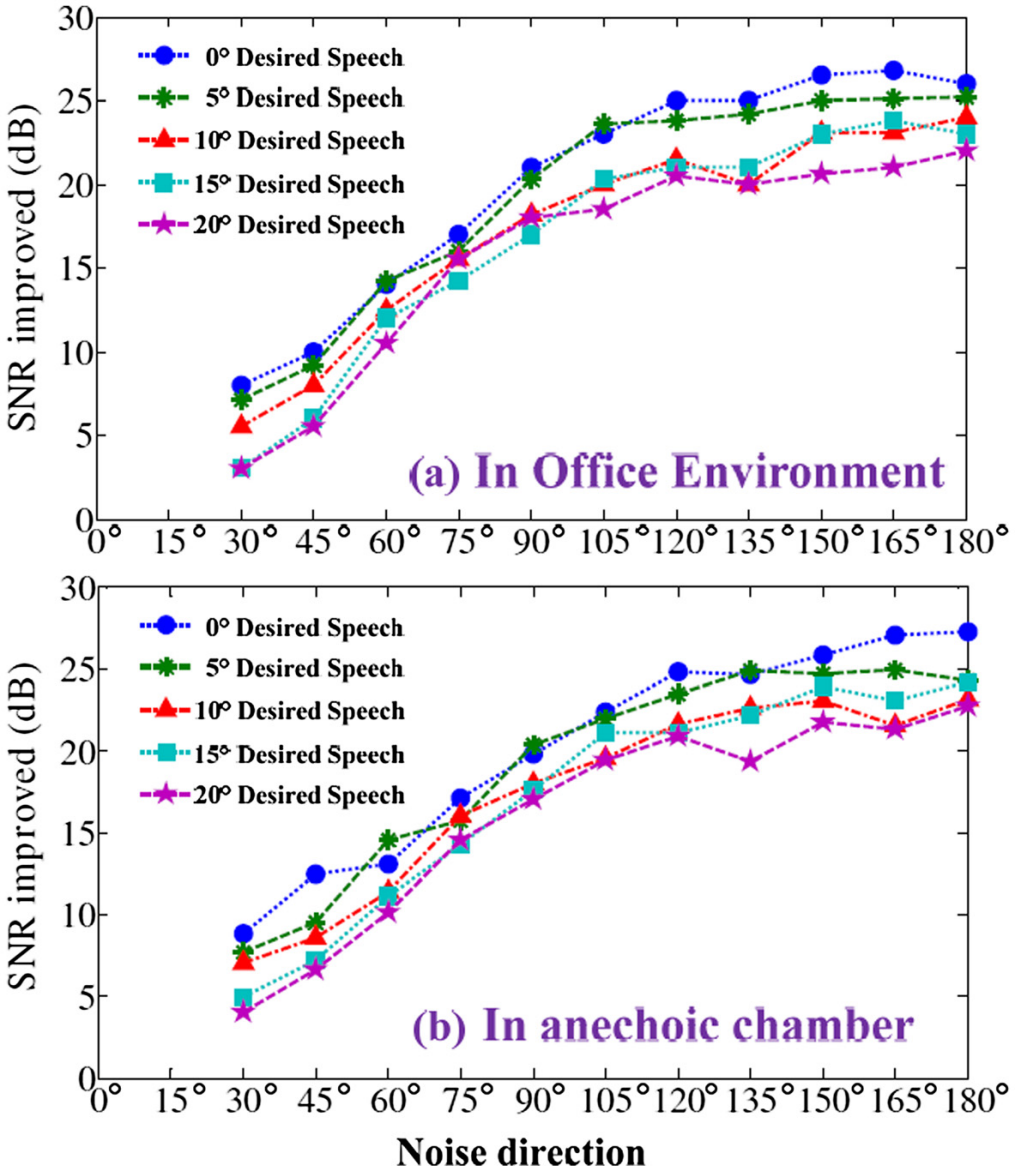
SNR improvement for head deviation. (a) in office environment, (b) in an anechoic chamber.

For actual CI devices, the dual microphones may not remain exactly collinear with the forward direction. Additionally, the head offset for CI users results in an orientation deviation of the desired speech. For daily face-to-face conversation, the microphone bias is primarily <20°. In this test, the orientation offset was 20°, and the ambient noise moved from 30° to 180° (the mirror-reversed orientation is between 180° and 330°), which covers the most probable range of head deviation and the noise direction. The desired speech (5° intervals) and the ambient noise (15° intervals) were played. The speech-enhancement results are plotted in Figure 9(a), in which the original input SNR was 0 dB.

Figure 9 describes the SNR improvement (in dB) for all situations in which the noise comes from 30° to 180° and the desired speech is played at the azimuth of 0°, 5°, 10°, 15°, or 20°. In the office environment with T_60_ = 450 ms, for a fixed speech direction, the improved SNR (Figure 9 (a)) was higher when the noise azimuth approached 180° (backward), and was lower when the noise approached the desired speech (forward). For the situation of speech deviation to the 0° azimuth, greater offset resulted in less SNR improvement. The plot also indicates that, for a speech deviation range of 0° to 20° and noise range of 60° to 180°, the SNR improvement was >10 dB. For a noise azimuth of 180° to 300°, the expected analogous and mirror-reversed result was obtained. For comparison, experiments were also carried out in an anechoic chamber (T_60_ = 100 ms), and the SNRs improvement for head deviation are plotted (Figure 9 (b)). A set of similar SNR results are obtained, with only 1 to 3 dB globally SNR increased in situation of anechoic environment. The room reverberation actually influences the algorithm performance, but in an acceptable constraint.

The prevalent algorithms can be seen as the beamformers to extract the desired signal from a certain direction while minimizing the output power of the ambient noise from another direction. These methods are advantageous in low speech distortion or high noise-reduction performance. Therefore, a compromise between signal distortion and noise suppression is needed. For spectrum estimation and subtraction based method, the distortion of the desired speech is unavoidable in our algorithm. To delineate the speech distortion, the speech distortion index is used [40-42], with the vector expression given in Eq. (24).

(24) $$v_{\mathrm{sd}}\left(h\right)=\frac{{\left(h_{1}-h\right)}^{T}R_{\mathrm{xx}}\left(h_{1}-h\right)}{h_{1}^{T}R_{\mathrm{xx}}h_{1}}$$

And the actual expression for calculation is given in Eq. (25).

(25) $$v_{\mathrm{sd}}\left(h\right)=\frac{\sum_{i=1}^{L}\frac{\lambda_{i}}{{\left(1+\lambda \right)}^{2}}b_{i1}^{2}}{\sum_{i=1}^{L}\lambda_{i}b_{i1}^{2}}$$

where *λ*_*i*_ is the element of diagonal matrix given in [42]. Actually, the speech distortion index presents the attenuation between speech power and the original clean speech.

**Figure 10:**
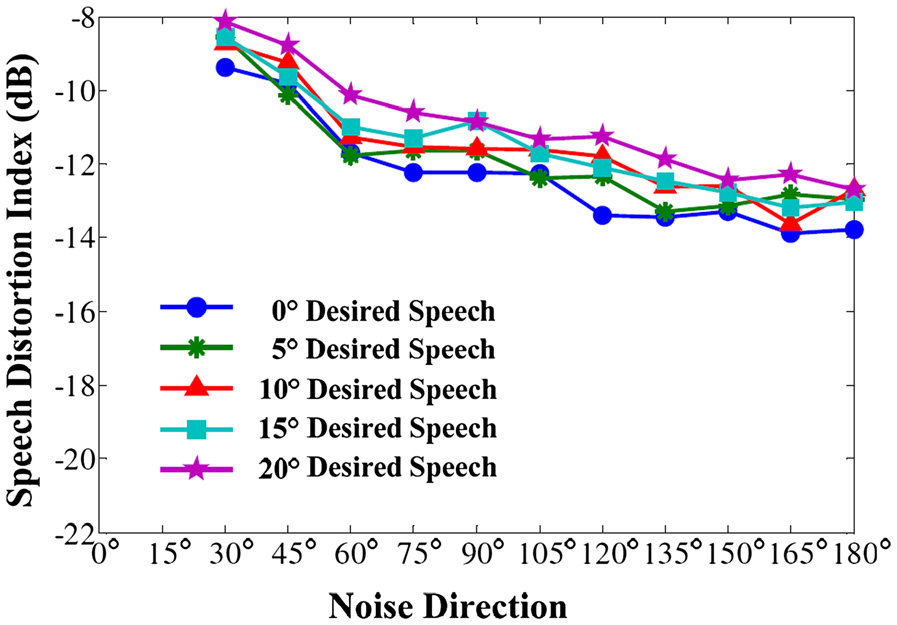
Speech distortion index for head deviation (in office environment).

And the distortion results are presented in Figure 10.

**Figure 11:**
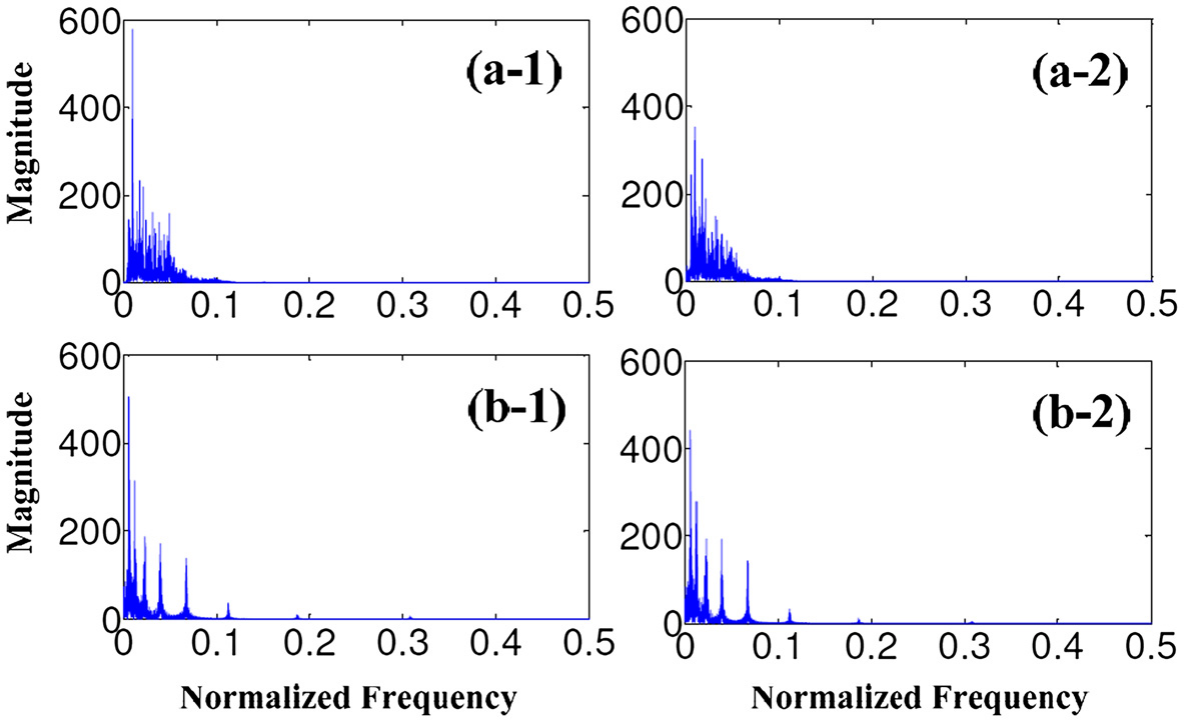
Signal spectrums of the original clean speech (a-1) and the enhanced speech (a-2), and the corresponding signal spectrums after the modulation of CI CIS strategy in (b-1) and (b-2).

Figure 10 depicts the speech distortion index (dB) for head deviation, corresponding the office-environment experiments also for all situations in which the noise comes from 30° to 180° and the desired speech is played at the azimuth from 0° to 20°. As expect, the speech distortion is noticeably large, ranging from −8 to −14 dB. It implies that our algorithm obtains high SNR but with a little bit large of speech distortion. Compared with other conventional methods in decreasing speech distortion, the proposed algorithm is not advantageous, or of great disadvantage compared with the time-domain beamformers (ANF etc.). But after the modulation of the CIS strategy in CI processor, with the signal envelope and signal information of the enhanced speech extracted and transferred to the CI electrode array, the signal distortion will be attenuated. For clear comparison, the signal magnitude spectrums are used to analyze the speech distortion, shown in Figure 11.

**Figure 12:**
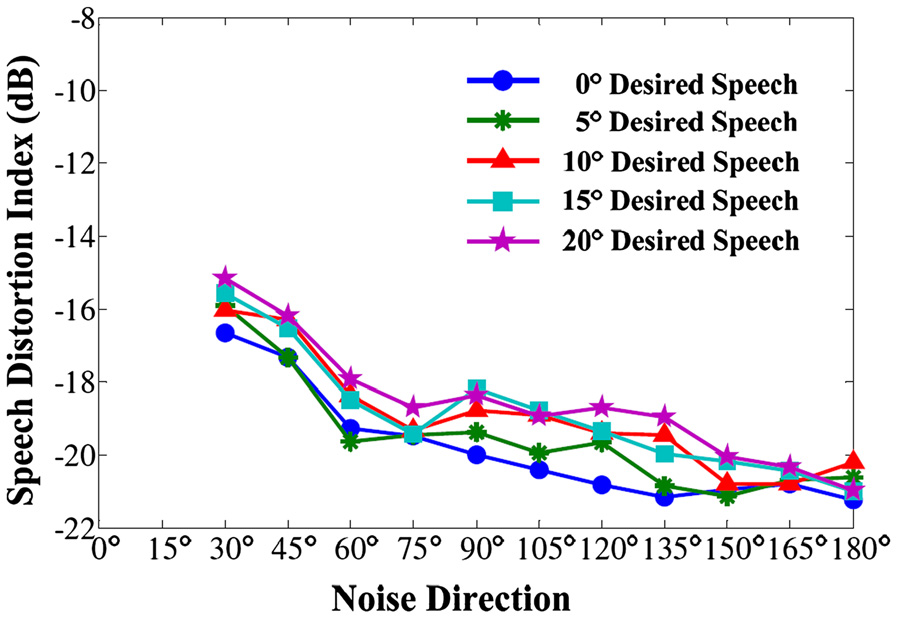
Speech distortion index for the enhanced signals after the CIS modulation (in office environment).

In Figure 11, panels a-1 and a-2 describe the signal spectrums of the original clean speech and the enhanced speech, and panels b-1 and b-2 are the corresponding spectrums after CIS modulation. Comparing the result in panel a-1 and a-2 (speech-enhancement result before the processing of CIS strategy) the spectrum difference is noticeable. But after the CIS processing (panels b-1 and b-2), with the signal energy concentrated in the central frequency of each sub-band in the CI filter bank, signal spectrums change to be approximately the same. These results imply that the final modulated signal to the CI electrodes will become low distorted. To further quantify the speech distortion for the enhanced signal after the CIS processing, another graph of the speech distortion index is plotted (Figure 12).

Figure 12 describes a graph of the speech distortion index for the enhancement of the desired speech after the CIS processing. The speech distortion indexes are sharply small, mainly between −18 and 21 dB. A smaller value of the speech distortion index means the desired signal is less distorted. Compared with figure 10, it prominently depicts a set of low speech distortion for the application of CI devices, which is in an acceptable range of signal distortion. For CI front-end signal acquisition, our algorithm is advantageous for a large amount of SNR improvement, but disadvantageous in a little bit of greater speech distortion when comparing with other low-distortion algorithms. However, for signal modulation and transmission by the CI CIS strategy, the speech distortion is sharply decreased.

These results, including the test of moving noise, head deviation (Figure 9) and algorithm evaluation of speech distortion (Figures 10, 11 and 12), indicate that the proposed algorithm is robust and flexible for CI speech-enhancement, with great SNR improvement and low speech distortion.

## Discussion

### Approximation of directivity coefficient

The approximation of the directivity coefficient in Eq. (15), corresponding to the noise azimuth *φ*, is important to algorithm performance. The hardware platform was also constructed for the experiments to evaluate the approximation. Firstly, the test was carried out in an anechoic chamber (T_60_ = 100 ms). The loudspeaker played music as the ambient noise at 90°, 180° and 270° orientations respectively. The target speech located at 0° orientation, with equal-power signal play from the loudspeaker (SNR = 0). The calculated orientations for the noise azimuth *φ* in Eq. (15) are 81°, 192° and 280°. The orientation error is about 10° for this situation. And the corresponding results are 77°, 171° and 285° respectively for the test in office environment (T_60_ = 450 ms), with about 15° errors. These errors is acceptable for the CI application, therefore, the directivity coefficient can be applied in the estimation of desired signal power spectrum in Eq. (14).

### Algorithm constraint for noise estimation

**Figure 13:**
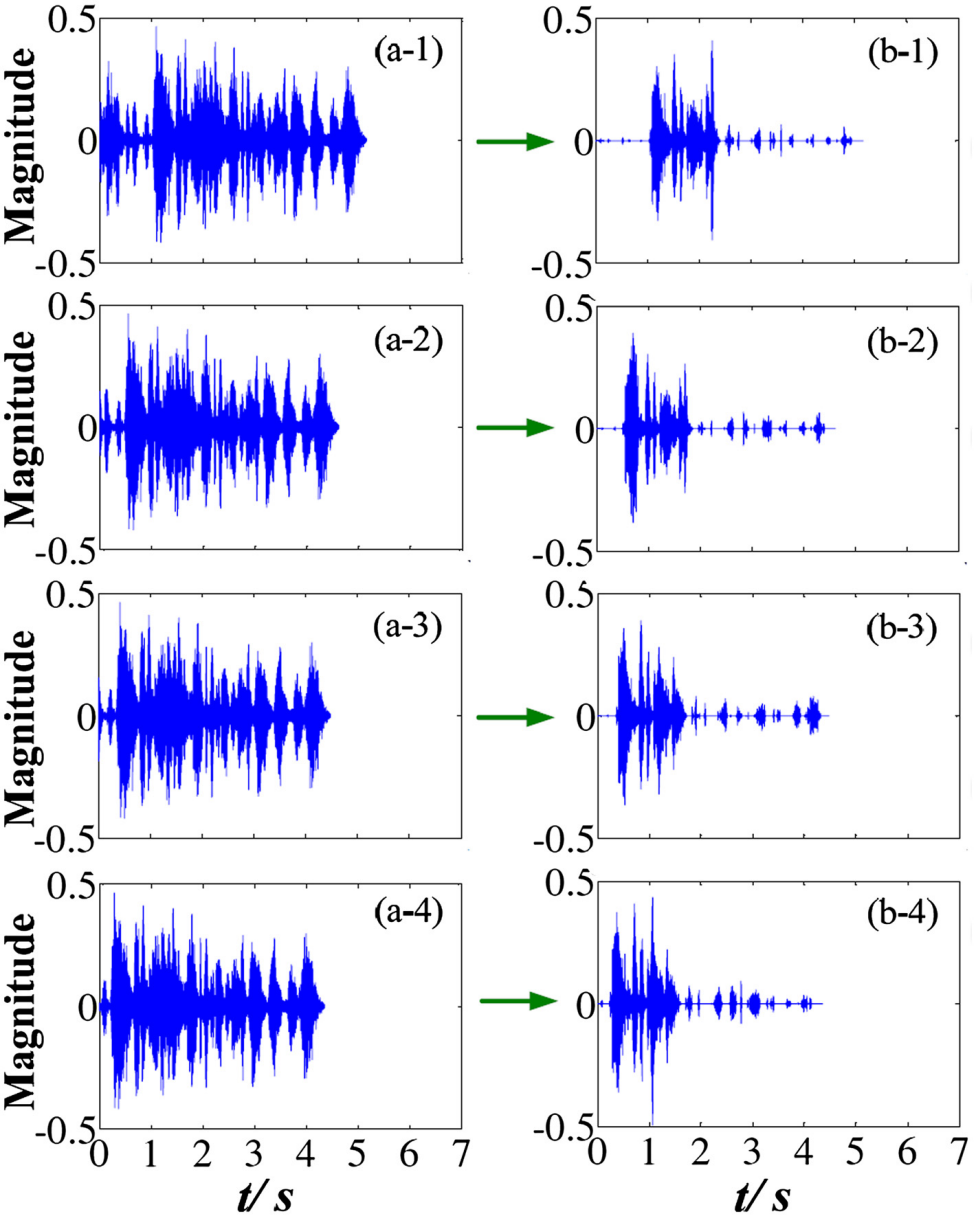
**Speech enhancement results for different lengths of the ambient-noise before the desired speech segment.** (**a-1**), (**a-2**), (**a-3**) and (**a-4**) are the situations of 1 s, 450 ms, 300 ms and 200 ms lengths of the noise-only interval before the speech segment respectively, and the corresponding speech-enhancement results are shown in (**b-1**), (**b-2**), (**b-3**) and (**b-4**).

As detailed analysis of the directivity coefficient in the aforementioned sections (seen in Figure 1, Eqs. (14) and (15)), the cepstrum method) was used for noise estimation. To simplify the test, as well as to be convenient for algorithm analysis and performance evaluation, the previous experiments use two long noise-only periods (about 3 seconds each) before and after the desired speech segment (about 1 second). However, the length of the ambient noise interval does not need as long as 3 seconds. For situations of shorter ambient noise, the results for speech enhancement are listed in Figure 13.

There are noise-only segments before and after the desired speech segment. In our algorithm, the anterior noise segment (before the desired speech) is used to estimate the directivity coefficient, and the length of which will influence the algorithm performance. For situations of the ambient noise with length no shorter than 450 ms (panels a-1 and a-2), the enhanced signals (panels b-1 and b-2) still remain great SNR improvement. For shorter length of ambient noise (panels a-3 and a-4), the SNR decreases noticeably (panels b-3 and b-4), as well as more speech distortion. To remain algorithm performance and low speech distortion in CIS modulation for CI devices, the minimal length of the noise-only period before the speech segment is about 0.5 second. This delaying time is acceptable for CI users in daily conversation. Specially, if we do not need a great SNR improvement, the length of the noise-only segment for pre-estimation can decrease to be about or less than 200 ms.

### Distortion in CI processing

The previous results indicate that the proposed algorithm introduced a bit larger distortion to the desired speech but with noticeable low distortion after the CI processing. This phenomenon may result from the speech processing strategy of CI devices. The CIS strategy is a speech processing strategy to extract the signal information from the time-domain envelope and then be transferred to the electrode array. This CIS speech strategy primarily includes the process of window-adding, frame-dividing, pre-emphasis, sub-band dividing, envelop extraction and signal compression. As the CIs only have several channels, correspond to a few specific stimulating rates, the extracted envelope may lose lots of signal information. In addition, in each band of the CI channel, only one frequency (correspond to the center frequency of the CI filter bank) is applied to modulate the desired signal. That is, a set of sinusoidal signals (only 16 or 24 for CI devices) are modulated by the corresponding envelope of the band-pass signals in the CI filter bank. Therefore, the single-frequency modulation processing seems to be a smoothness process to reduce the distortion. For example, taking the distortion in the band of [1653, 1960] (channel 9) into account. If the 1700 Hz signal is strengthened but the 1900 Hz signal is weakened, the distortion will be introduced in this band. But after the CI modulation, both frequencies in this band, the center frequency 1807 Hz sinusoidal signal is applied to modulate the envelope based on the whole band energy. Therefore, the difference between the same bands will be smoothed, and the speech distortion after CI processing may come from the difference between different bands. Consequently, the CI speech strategy can reduce speech distortion and more aggressive algorithm can be applied in the CI application.

### Algorithm performance

The proposed algorithm is an aggressive noise-suppression method with high SNR improved but a bit large distortion. But the distortion can be reduced in the CI processing and we can obtain excellent performance. The prevalent Frost algorithm based methods, such as linearly constrained minimum-variance and MINT algorithms, can suppress the noise with less signal distortion. These methods use iterative-adaptive technique to update the filter coefficients by gradient estimation and are advantageous in minimizing the ambient noise with low or no distortion for desired signal. Whereas, when the moving noise (or in the situation that the noise changes its azimuth) is present, these methods will weaken their noise-reduction performance. To obtain the optimal filter coefficients, if the algorithm does not reconvergent at the beginning or too slow to update the new coefficients, the noise will leakage and the desired performance will be attenuated. Other methods, MVDR and the binaural frequency-domain minimum-variance algorithm etc., present effective ways for noise suppression. Though these algorithms can converge more quickly, they will also cause noise leakage and the algorithm performance will be weakened. The approximation of directivity coefficient in our algorithm is tested, and about 10° error can be found. The directivity coefficient estimation is accurate enough to separate the desired speech and the ambient noise. For the trade-off between SNR improvement and speech distortion, the prevalent optimal-filter methods minimize the speech distortion while guarantying a certainly level of SNR improvement or maximize the SNR improvement while guarantying a certainly level of speech distortion. So it is hard for these algorithms to obtain both high SNR and low signal distortion. However, in the application of cochlear implant, the CI processing helps to obtain excellent speech enhancement while guarantying low speech distortion in the proposed algorithm.

## Conclusions

The proposed speech-enhancement algorithm based on a dual-channel microphone array and spectral estimation technique aims to suppress the directional noise and improve the speech recognition of CI devices. A hardware platform was constructed and the experiments were carried out in an office to evaluate the algorithm performance in a real working environment for CI users. The experimental results indicated the excellent algorithm performance for speech enhancement. For stationary and moving noises, in orientations from lateral to rear, the improvements in SNR were 20 and 15 dB, respectively. For the situation of ± 20° speech deviation and a broad range of noise azimuths from 60° to 300°, SNR improvement of >10 dB was maintained. Also, the speech distortion was very low when evaluating the modulated signal in CIS processing. The proposed algorithm is robust to mobile noise and signal orientation deviation and is applicable to the improvement of the front-end signal acquisition and speech recognition for CI devices.

## Abbreviations

CI: Cochlear implant; SNR: Signal to noise ratio; FDM: First-order differential microphone; ANF: Adaptive null-forming method; MINT: Multiple input/output inverse method; MVDR: Minimum-variance distortionless-response technique; Maxflat: Maximal flat; FIR: Finite impulse response; CIS: Continuous interleaved sampling strategy; ACE: Advanced combined encoder strategy.

## Competing interests

The authors declare that they have no competing interests.

## Authors' contributions

YC initiated and conceived the algorithm, designed the hardware system and experiments and analyzed the data. QG is the corresponding author. This study originated from QG’s idea and problems were solved under her directions. QG drafted this paper’s manuscript, including content and overall arrangement. QG was also responsible for revising this manuscript. All authors read and approved the final manuscript.
